# Negative Predictors of Tooth Extraction in the Management of Odontogenic Sinusitis in a Japanese Patient Population: A Retrospective Study

**DOI:** 10.1055/s-0044-1791492

**Published:** 2025-01-10

**Authors:** Kazuhiro Hirasawa, Koji Otsuka, Renako Tomaru, Naoki Ikehata, Kiyoaki Tsukahara

**Affiliations:** 1 Department of Otorhinolaryngology, Toda Central General Hospital, Toda-shi, Japan; 2 Department of Otorhinolaryngology, Head and Neck Surgery, Tokyo Medical University, Shinjuku-ku, Tokyo, Japan; 3 Kampo Medicine Center, Tokyo Medical University Hospital, Shinjuku-ku, Tokyo, Japan; 4 Department of Otorhinolaryngology, Tokyo Medical University Ibaraki Medical Center, Inashiki-gun, Ibaraki, Japan; 5 Department of Dental and Oral Surgery, Tokyo Medical University Ibaraki Medical Center, Inashiki-gun, Ibaraki, Japan; 6 Department of Oral and Maxillofacial Surgery, Tokyo Medical University, Shinjuku-ku, Tokyo, Japan

**Keywords:** odontogenic sinusitis, tooth extraction, polyps, Lund-Mackay score, apical lesions

## Abstract

**Introduction** There are no clear guidelines for deciding between endoscopic sinus surgery and tooth extraction for the treatment of odontogenic sinusitis. Furthermore, tooth extraction does not necessarily improve sinusitis and eventually results in additional endoscopic sinus surgery.

**Objective** The present study aimed to retrospectively investigate negative predictive factors of tooth extraction for odontogenic sinusitis.

**Methods** In total, 22 patients with odontogenic sinusitis, who underwent tooth extraction between April 2017 and March 2021, were included. The patients were divided into the improved (n = 15) and non-improved (n = 7) groups. Subsequently, the two groups were compared.

**Results** A higher percentage of patients in the non-improved group had polyps in the middle nasal meatus ( *p* = 0.0008), higher Lund-Mackay score (LMS) ( *p* = 0.0008), and apical lesions penetrating the maxillary sinus ( *p* = 0.113). Patients with middle nasal meatus polyps, with LMS ≥ 7, or with a combination of apical lesions penetrating the maxillary sinus and LMS ≥ 5, were less likely to see improvement in sinusitis with tooth extraction.

**Conclusion** Tooth extraction as the initial intervention for odontogenic sinusitis presents a higher risk of failure, particularly in cases in which polyps are present in the middle nasal meatus, with LMS ≥ 7, or with a combination of apical lesions penetrating the maxillary sinus and LMS ≥ 5.

## Introduction

Odontogenic sinusitis is a condition in which dental infections spread to the maxillary and other sinuses, and it is a well-recognized condition in otolaryngology and oral surgery. There are no clear guidelines for the treatment of choice for odontogenic sinusitis. 1 Opinions regarding the optimal treatment approach for odontogenic sinusitis vary. Some suggest that tooth extraction should be performed first, 2 while others recommend a combined approach of tooth extraction and endoscopic sinus surgery (ESS). 3 4 However, in Japan, Sato et al. suggest an ESS-first approach to preserve as much of the affected tooth as possible. 5 6 7 Consistent improvement in sinusitis through tooth extraction is not guaranteed and may ultimately require ESS. 8 On the other hand, patients may prefer to undergo tooth extraction first because it is less invasive than ESS. Therefore, when deciding on the optimal treatment strategy, it is necessary to determine whether sinusitis can be controlled by tooth extraction alone.

It is expected that mild odontogenic sinusitis can be controlled by isolated tooth extraction. 9 However, few reports have examined this in detail. Only one worldwide report on the subject has been identified, as reported by Mattos et al. 8 As per our current understanding, no Japanese work has been published prior to the present one. In this study, we retrospectively collected cases of Japanese patients who underwent tooth extraction for odontogenic sinusitis and analyzed negative predictive factors of tooth extraction for odontogenic sinusitis.

## Methods

### Study Patients

**Fig. 1:**
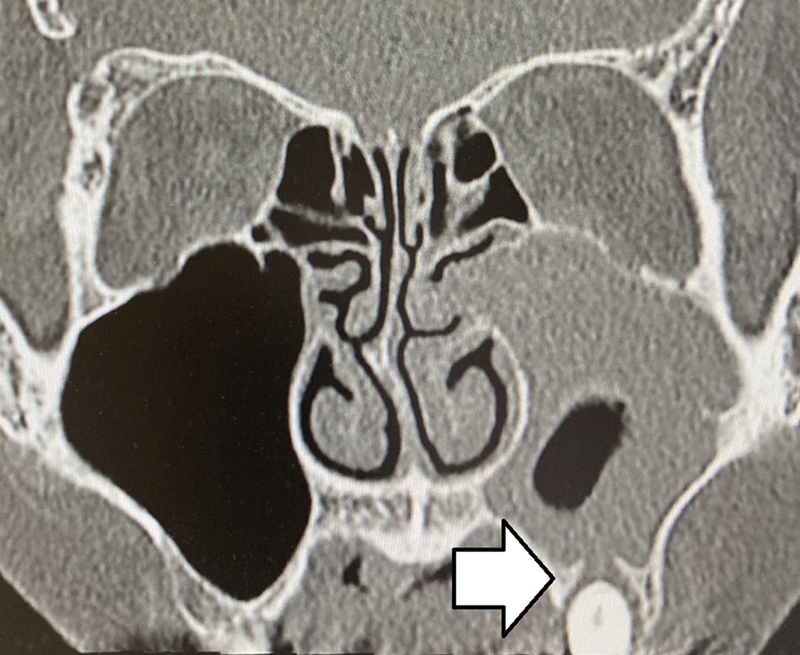
Computed tomography image of odontogenic sinusitis. Unilateral sinusitis with a root apex lesion is observed (white arrow).

In the present study, odontogenic sinusitis was defined as a unilateral, periapical lesion observed on a computed tomography (CT) scan, classified as odontogenic by an oral surgeon in our department ( Fig. 1 ). Between April 2017 and March 2021, 26 patients visited the Tokyo Medical University Ibaraki Medical Center and underwent tooth extraction for odontogenic sinusitis. Of these, 22 patients were included, while 4 were excluded: 3 for absence of follow-up, and 1 for co-occurrence of fungal sinusitis. The included patients were divided into 2 groups: the improved group (n = 15) and the non-improved group (n = 7). The improved group included patients who only required isolated tooth extraction and showed the resolution of clinical symptoms, improvement of intranasal endoscopic findings, or disappearance of sinus shadows on CT scans. The non-improved group included patients who required subsequent ESS. The primary variables analyzed were the presence of polyps in the middle nasal meatus, diabetes mellitus, ipsilateral nasal septum deviation, Lund-Mackay score (LMS), 10 and apical lesion penetration into the maxillary sinus. Age and gender were also compared between the two groups in terms of patient characteristics.

The current retrospective study was conducted in compliance with the Declaration of Helsinki and ethical guidelines for medical research targeting humans. It was approved by the Tokyo Medical University Medical Ethics Review Board (approval number: TS2022-0337). Informed consent was unnecessary due to the retrospective nature of the study.

### Statistical Analysis

The Mann-Whitney U test was used to compare the ages and LMSs of the two groups. The Fisher exact test was used for comparison of the remaining variables. A *p* -value < 0.05 was considered statistically significant, with a 2-tailed test being used for gender, and a 1-tailed test employed for the remaining variables.

## Results

### Patients' Characteristics ( Table 1 )

**Table 1:** Patients' characteristics

	Improved group (n = 15)	Non-improved group (n = 7)	*p* -value
**Age (years)**	**60 ± 15.4 (32–83)**	**65 ± 12.9 (48–87)**	**0.244**
**Gender** **Men** **Women**	**9** **6**	**3** **4**	**0.65**

There were 15 and 7 patients in the improved and non-improved groups, respectively. There were no significant differences in age or gender between the two groups. The age distribution was 32 to 83 years (average age: 57.4 years) and 48 to 87 years (average age: 66.0 years) in the improved and non-improved groups, respectively. The improved group included 9 men and 6 women, while the non-improved group included 3 men and 4 women.

### Presence of Polyps in the Middle Nasal Meatus ( Table 2 )

**Table 2:** Comparison between the improved group and non-improved groups

Clinical Finding	Improved group (n = 15)	Non-improved group (n = 7)	*p* -value
Middle nasal meatal polyps	0 case	5 cases	0.0008*
**Lund-Mackay score (LMS)** **2** **3** **4** **5** **6** **7** **8**	**3 cases** **6 cases** **1 case** **2 cases** **3 cases** **-** **-**	**-** **-** **-** **1 case** **1 case** **3 cases** **2 cases**	0.0008*
**Apical-lesion penetration into the maxillary sinus**	**5 cases**	**5 cases**	**0.113**
**Combination of** **apical-lesion penetration into the maxillary sinus** **and LMS ≥ 5**	**0 case**	**5 cases**	0.0008*
**Diabetes mellitus**	**2 cases**	**1 case**	**0.70**
**Ipsilateral nasal-septum deviation**	**7 cases**	**3 cases**	**0.62**

Note: *, significantly different.

The incidence of polyps in the middle nasal meatus was significantly higher in the non-improved group compared with the improved group ( *p* < 0.05). Five patients (71.4%) in the non-improved group had polyps, compared to 0 patients in the improved group.

### LMS ( Table 2 )

The LMS for the improved group was 2, 3, 4, 5, and 6 in 3, 6, 1, 2, and 3 cases, respectively, with an average score of 3.73. For the non-improved group, the LMS was 5, 6, 7, and 8 in 1, 1, 3, and 2 cases, respectively, with an average score of 6.86. The LMS was significantly higher in the non-improved group ( *p* < 0.05). Furthermore, all cases with LMS ≥ 7 did not improve.

### Presence of Penetration of Apical Lesion into the Maxillary Sinus ( Table 2 )

Although a higher percentage of patients in the non-improved group had apical lesion penetration into the maxillary sinus (71.4% vs 33.3%), this difference was not statistically significant.

### Presence of both Apical-Lesion Penetration into the Maxillary Sinus and LMS ≥ 5 ( Table 2 )

The percentage of patients with both apical-lesion penetration of the maxillary sinus and LMS ≥ 5 was significantly different between the groups (71.4% in the non-improved group, 0% in the improved group, *p* < 0.05).

### Presence of Diabetes Mellitus ( Table 2 )

No significant difference was found in the incidence of diabetes mellitus (13.3% in the improved group and 14.3% in the non-improved group).

### Presence of Ipsilateral Nasal Septal Deviation ( Table 2 )

Although 7 patients (46.7%) in the improved group had ipsilateral nasal septum deviation compared to only 3 (42.9%) in the non-improved group, this was not statistically significant.

## Discussion

### Diagnostic Criteria for Odontogenic Sinusitis

The diagnostic criteria for odontogenic sinusitis are not well established; however, some reports suggest that many cases of unilateral sinusitis are odontogenic. 11 12 Computed tomography scans have revealed that approximately two thirds of cases of odontogenic sinusitis have associated periapical-periradicular infection. 13 Therefore, in this study, we included cases in which a unilateral periapical lesion was observed on CT.

### Reduced Tooth Extraction Success in Presence of Nasal Polyps

Resolution of odontogenic sinusitis occurs more frequently compared to cases of general chronic sinusitis. 14 Facilitating ventilation and drainage is critical to this process. 5 However, if polyps grow in the middle nasal meatus, ventilation and excretion are more difficult, reducing the success of tooth extraction for treatment of odontogenic sinusitis. In our study, tooth extraction did not resolve the sinusitis in all five patients with polyps in the middle nasal meatus.

### Reduced Tooth Extraction Success with Higher LMS

The LMS was significantly higher in the non-improved group, and especially in cases with LMS ≥ 7, which showed no improvement. Mattos et al. 8 and Yoo et al. 15 also reported less improvement in cases with higher LMS, which agrees with our study results. We previously reported that in deep neck cellulitis, the success of conservative treatment decreased with a higher number of infected spaces. 16 Similarly, for odontogenic sinusitis, we report here that there was a significant decrease in the success of tooth extraction when LMS was high.

### Reduced Tooth Extraction Success with Combination of LMS ≥ 5 and Presence of Apical-Lesion Penetration into the Maxillary Sinus

Apical-lesion penetration into the maxillary sinus alone, thus facilitating the spread of inflammation and potentially worsening the disease, 17 was not associated with a significant decrease in tooth extraction success rate in our study. However, when combining apical-lesion penetration with LMS ≥ 5, there was a significant decline in success rate of tooth extraction. Therefore, the combination of apical-lesion penetration into the maxillary sinus and LMS ≥ 5 is a valid negative predictor of success for isolated tooth extraction.

### Diabetes Mellitus and Ipsilateral Nasal Septal Deviation

Diabetes mellitus causes a decline in immune cell function and diminishes the resistance to infection. 18 Though our study did not find a significant difference in the improvement rate between patients with or without diabetes, the sample size was small (three cases). Further research with a larger sample size is needed to better understand this relationship.

Nasal septum deviation, like the presence of nasal polyps, narrows the ostiomeatal complex (OMC), constricting ventilation and excretion routes and potentially leading to more persistent sinus inflammation. 19 However, ipsilateral nasal septal deviation did not lead to a significant difference in the improvement rate in our study while the presence of nasal polyps did. A previous study has found no relation between nasal septum deviation and rhinosinusitis, 20 and our results were consistent with these.

### Treatment Strategy for Odontogenic Sinusitis Based on the Above Results

Endoscopic Sinus Surgery should be considered before tooth extraction in cases of odontogenic sinusitis with polyps in the middle nasal meatus, with LMS ≥ 7, or with a combination of apical lesions penetrating the maxillary sinus and LMS of 5 or 6. In such cases, the affected tooth may be preserved. In other cases, the optimal approach is less clear. Clinicians should discuss with patients the risks and benefits of starting with either tooth extraction or ESS. Based on the study by Craig et al., 21 we consider it ESS preferable when the symptoms caused by sinusitis are painful and need to be resolved immediately.

In addition, it is noteworthy that preserving a tooth with marginal periodontitis and significant movement is inherently challenging. 5 In such cases, tooth extraction is mandatory. Although the timing of tooth extraction was not verified in this study, considering the findings of the study by Yoo et al., 15 it may be desirable to proceed with ESS as soon as possible to eliminate inflammation before tooth extraction to prevent maxillary fistula.

The limitations of the present study include a small sample size, and inability to generalize the findings to different populations. Additionally, there is potential for selection bias as all the patients were recruited from a single institution.

## Conclusions

This study explored negative predictors of successful tooth extraction for odontogenic sinusitis. Endoscopic sinus surgery should be considered the first-line treatment in cases with polyps in the middle nasal meatus, with LMS ≥ 7, or with the combination of apical lesions penetrating the maxillary sinus and LMS of 5 or 6. In such cases, the affected tooth may be preserved. The optimal first approach in other cases is less clear. Clinicians should include patients in the decision-making process, based on the risks and benefits of starting with tooth extraction or ESS.
